# Evolution of Alternative Splicing Regulation: Changes in Predicted Exonic Splicing Regulators Are Not Associated with Changes in Alternative Splicing Levels in Primates

**DOI:** 10.1371/journal.pone.0005800

**Published:** 2009-06-04

**Authors:** Manuel Irimia, Jakob Lewin Rukov, Scott William Roy

**Affiliations:** 1 Departament de Genètica, Facultat de Biologia, Universitat de Barcelona, Barcelona, Spain; 2 Molecular Evolution Group, Department of Biology, University of Copenhagen, Copenhagen, Denmark; 3 National Center for Biotechnology Information, National Library of Medicine, National Institutes of Health, Bethesda, Maryland, United States of America; Centre de Regulació Genòmica, Spain

## Abstract

Alternative splicing is tightly regulated in a spatio-temporal and quantitative manner. This regulation is achieved by a complex interplay between spliceosomal (*trans*) factors that bind to different sequence (*cis*) elements. *cis*-elements reside in both introns and exons and may either enhance or silence splicing. Differential combinations of *cis*-elements allows for a huge diversity of overall splicing signals, together comprising a complex ‘splicing code’. Many *cis*-elements have been identified, and their effects on exon inclusion levels demonstrated in reporter systems. However, the impact of interspecific differences in these elements on the evolution of alternative splicing levels has not yet been investigated at genomic level. Here we study the effect of interspecific differences in predicted exonic splicing regulators (ESRs) on exon inclusion levels in human and chimpanzee. For this purpose, we compiled and studied comprehensive datasets of predicted ESRs, identified by several computational and experimental approaches, as well as microarray data for changes in alternative splicing levels between human and chimpanzee. Surprisingly, we found no association between changes in predicted ESRs and changes in alternative splicing levels. This observation holds across different ESR exon positions, exon lengths, and 5′ splice site strengths. We suggest that this lack of association is mainly due to the great importance of context for ESR functionality: many ESR-like motifs in primates may have little or no effect on splicing, and thus interspecific changes at short-time scales may primarily occur in these effectively neutral ESRs. These results underscore the difficulties of using current computational ESR prediction algorithms to identify truly functionally important motifs, and provide a cautionary tale for studies of the effect of SNPs on splicing in human disease.

## Introduction

Alternative splicing (AS) generates multiple transcripts from the same gene by differential splicing of introns, thereby increasing transcriptome and proteome diversity [1]. Between 40–60% of all human genes [2]–[5] and up to 95% of multi-exon genes [6]–[8] are estimated to be alternatively spliced, and similar fractions have been estimated for other vertebrate species [9]. However, the portion of AS that is in fact functional remains unknown [10], [11]. Multiple studies have shown that alternatively spliced exons are less conserved than constitutively spliced ones, suggesting that much alternative splicing may not be functional (reviewed in [12], [13]). On the other hand, expression of many alternatively spliced exons is highly regulated through development, including the precise regulation of exon inclusion levels (i.e. the fraction of transcripts from a given locus that include an exon; [14]–[16]). Accordingly, several studies have shown that the precise regulation of AS is crucial for proper gene function (e.g. [17]–[19]), thus evolutionary changes in AS regulation are likely to affect phenotype.

Several recent comparative studies have probed AS regulation. Calarco *et al.* identified a subset of alternatively spliced exons with varying inclusion levels between humans and chimpanzees, based on quantitative microarray profiling [15]. The number of genes showing such changes in AS is comparable to previous estimates of the total number of genes that show differences in transcript level [20], [21]. This suggests that changes in regulation of the two processes–transcription and alternative splicing–make similar contributions to phenotypic differences between humans and chimpanzees. Interestingly, the two types of change are not significantly associated (that is, genes showing one type of change are not more likely to show the other), suggesting that the two types of changes may act largely independently.

In recent years, major progress has been made in understanding how (alternative) splicing is regulated. Vertebrate exons typically comprise only a minority of pre-mRNA transcript length, requiring accurate recognition of short exonic ‘islands’ in a ‘sea’ of intronic sequence [22]. This recognition is achieved by the binding of spliceosomal components (*trans*-factors) to a diverse array of intronic and exonic splicing sequence elements (*cis*-elements). The first layer of sequence signals consist of canonical splicing motifs including intronic splice sites, branch point and the polypyrimidine tract, which are recognized by the core spliceosomal components [23], [24]. These elements together are estimated to provide around 50% of the information necessary for exon recognition and intron splicing [25]. The remaining information is provided by a second, more complex, layer of *cis* elements. These elements are motifs located in exons and/or introns that act as splicing enhancers or silencers, and are important in regulating both constitutive and alternative splicing [26].

The best studied elements are those located in exons, called exonic splicing regulators (ESRs). These can either enhance (exonic splicing enhancers or ESEs) or reduce (exonic splicing silencers or ESSs) splicing at nearby splice sites. ESEs and ESSs function by recruiting *trans* splicing factors, often SR proteins and hnRNPs, respectively, that either promote or inhibit spliceosome assembly [26]–[28]. Using combinations of computational and experimental approaches, different research groups have identified many putative ESEs and ESSs and demonstrated their ability to modify exon inclusion levels, either by insertion of ESRs into reporter minigenes, or by mutational disruption of ESRs in naturally occurring exons [29]–[36]. ESRs occur in exons in different combinations, allowing for subtle control of individual splicing [37], [38], and together constitute a complex ‘splicing code’ [26], [39]. While much has been learned about the functioning of the splicing code in humans, the effects of changes in ESRs through primate evolution has not been explored at a large scale.

Here, we explore two basic predictions of the splicing code model. First, evolutionary changes in ESRs should lead to changes in AS exon inclusion levels. Second, the direction of these changes (i.e. increase or decrease in inclusion level) should be readily predictable from the specific change (e.g., disruption of an ESE should lead to decreased inclusion levels). We used quantitative microarray data to test these predictions for the evolution of AS expression levels in human and chimpanzee. Surprisingly, for all available ESR datasets, we find that changes in *cis*-elements are not associated with AS variations between the two species. This lack of association holds for ESRs located at different positions of the exons, and for different exon lengths and splice site strengths. We suggest that this lack of association is due to most changes in ESRs during recent primate evolution occurred in ESR-like motifs that are non-functional, due to their specific genetic/cellular context. These results thus attest to limitations of the current splicing code model in predicting AS evolution from a genome-wide perspective, and urge caution in the use of current ESR-prediction algorithms alone for identification of exonic motifs that are truly important for splicing.

## Results

### ESR density and change in alternatively spliced exons

We studied ESR motif composition in 1845 alternatively spliced exons conserved between human and chimpanzee. We used three different ESR datasets from previous studies [29], [30], [40], and a consensus dataset (consisting of ESR motifs contained in all three datasets, C-dataset). The observed ESR density was high, ranging 10.3 to 43.5 ESRs per 100 nucleotides, depending on the dataset (datasets differ considerably in total number of predicted ESRs, see Methods) (Table 1). Similarly, a high fraction (57.4% to 87.5%, depending on the ESR dataset) of exonic nucleotides were part of at least one ESR hexamer, indicating that predicted ESRs are widely distributed across exons and that a very large proportion of exonic sequence might potentially impact splicing regulation (Table 1).

**Table 1:** Table caption to follow.

	K-dataset			S-dataset			G-dataset	C-dataset	non-ESR
	ESE	ESS	all ESR	ESE	ESS	all ESR	ESR	ESR	
total count	39134	9560	48694	77717	21180	98897	23434	9394	110164
# changes	686	206	892	1521	453	1974	437	138	3056
%change	1.75	2.15	1.83	1.96	2.14	2.00	1.86	1.47	2.77
ESRs per 100 nt	17.22	4.21	21.43	34.20	9.32	43.53	10.31	4.13	-
% ESR-involved nt	50.54	18.60	62.18	73.50	30.82	87.51	51.43	22.94	-

Consistent with previous studies (e.g. Ke et al. 2008), we found a lower rate of change in predictive ESR motifs relative to other exonic sequence. The fraction of predicted ESR hexamers experiencing change between human and chimpanzee is low, ranging from 1.9–2.0%, compared to 2.8% of change of non-ESR hexamers (Table 1). Similarly, only 16 (0.87%) of exon pairs show changes in 5′ss strength between the two species, with changes in C.V. score higher than 5. In general, the average degree of nucleotide change in studied exonic sequences between the two species was very low (0.0041); out of the total 1845 studied exons, 1275 (69.10%) showed no changes between the two species, 381 (20.65%) had a single nucleotide change, 115 (6.23%) showed 2 changes, and 74 (4.01%) had more than two changes, minimizing the occurrence of potentially compensatory changes in our dataset.

### Changes in ESR composition are not associated with variation in AS inclusion level in primates

We here investigate the hypothesis that sequence changes in predicted ESRs are associated with changes in inclusion levels of alternatively spliced exons. We used various cutoffs for an exon to be considered to exhibit significant change in inclusion level between species: >20% difference in inclusion level between species, >25% or >30%.

**Figure 1:**
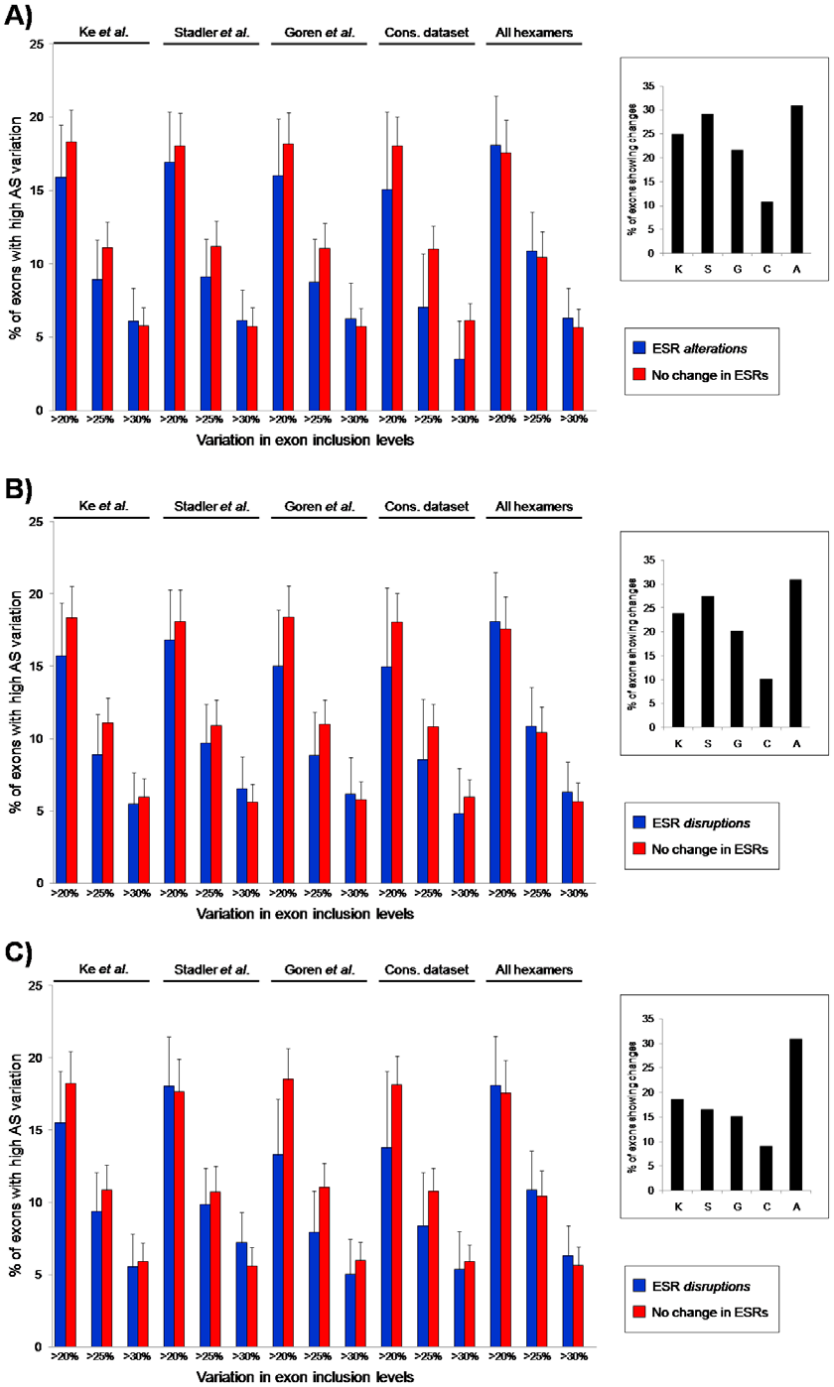
eneral lack of association between ESR changes and AS variation. In blue, percentage of exons with (A) ESR-altering changes between human and chimpanzee, (B) ESR-disrupting changes, or (C) ESR-disrupting changes in all overlapping hexamers, for the different datasets, that show high level of exon inclusion level interspecific changes, for different cutoffs (y-axis, >20% difference in inclusion levels, >25% or >30%). In red, the percentage of exons without changes at predicted ESRs showing high level of AS variation. The similar percentage of exons with high AS variation indicates a lack of general association between changes in predicted ESRs and AS levels. Right-hand side panels show the percentage of the all exons that have changes in ESRs for the different available datasets.

For all available ESR datasets (see Methods), exons with interspecific sequence changes within predicted ESRs (i.e. ‘ESR-altering’ changes; see Methods) are not more likely to exhibit interspecific differences in inclusion level than other exons (Figure 1A). Also consistent with previous results [15], we observed no association between sequence change overall (‘All hexamers’ in Figure 1A, essentially comparing identical with non-identical exons).

All changes within ESRs are not equivalent, however. For instance, a change within an ESE may yield a non-ESR motif, or it may yield an ESS, or it may yield a different ESE sequence. In the last case, the change may not change the splicing pattern much or at all [30], thus it is necessary to distinguish between types of changes. Studying only exons with changes that disrupt ESRs (see Methods), we obtained similar results (Figure 1B). Similar results were also obtained using a stricter criterion for ESR disrupting changes, that is, if a basepair change introduces a ESR in one species, none of the corresponding 6 overlapping hexamers in the other species can be an ESR [40](Figure 1C).

**Figure 2:**
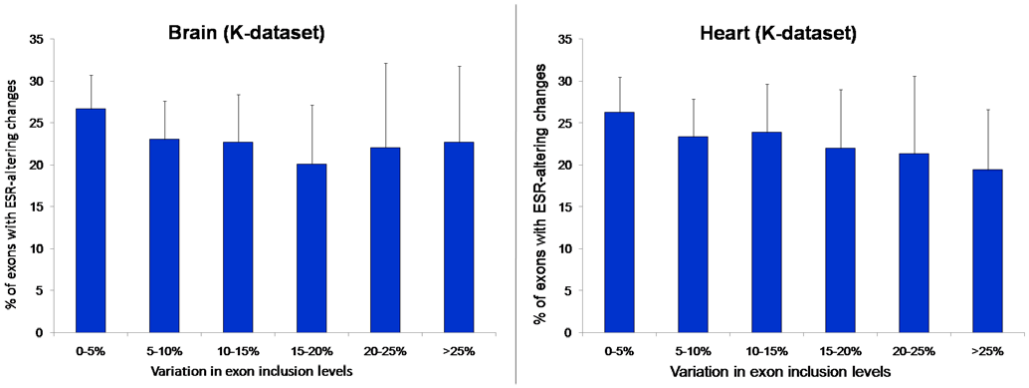
ercentage of exons showing ESR-altering changes for different groups of AS level variation for brain context (left) and heart (right). These results correspond to ESRs from Ke *et al.*'s dataset, and they are similar for the other available dataset and overall nucleotide change (data not shown).

The general lack of association between changes in predicted ESRs and in inclusion level held when the data was analyzed from a variety of perspectives, including considering each tissue separately, and considering predicted ESEs and ESSs separately (see Figures S1, S2 and S3). To further study the data, for each tissue we divided exons into subgroups according to their observed AS variation levels (0–5%, 5–10%, 10–15%, 15–20%, 20–25% and >25% difference in inclusion levels). For each of these levels of AS variation, we studied the fraction of exons that showed changes in ESR composition, finding similar values for all subgroups in both tissues and for all studied datasets (Figure 2).

Restricting the analysis to changes in experimentally determined consensus motifs bound by well-known *trans*-factors (SC35, SRp40, SRp55, SF2/ASF and hnRNPA1, see Methods) also showed no association (Table 2), although the number of changes observed in these motifs is too small to reach confident conclusions.

**Table 2:** Table caption to follow.

		Exons with >20% shift in inclusion	Total exons	% exons	S.E.
**SF2**	Motif disruption	1	15	6.67	13.03
**(**crsmsgw)	No disruption	326	1830	17.81	1.89
**SC35**	Motif disruption	1	14	7.14	13.96
**(**gryymcyr)	No disruption	326	1831	17.80	1.89
**SRp40**	Motif disruption	4	25	16.00	15.39
**(**yywcwsg)	No disruption	323	1820	17.75	1.89
**SRp55**	Motif disruption	4	33	12.12	11.77
**(**yrcrkm)	No disruption	323	1812	17.83	1.90
**hnRNP**	Motif disruption	1	1	100.00	–
(tagggw)	No disruption	326	1844	17.68	1.87

Finally, we also found no correlation between the density and total number of ESRs in an exon and the interspecific difference in inclusion levels (R^2^ ranged from 0.001 and 0 for the different ESR datasets and tissues).

### Lack of association between ESR change and AS level variation holds across ESR exonic position, exon length, and intron splice site strength

**Figure 3:**
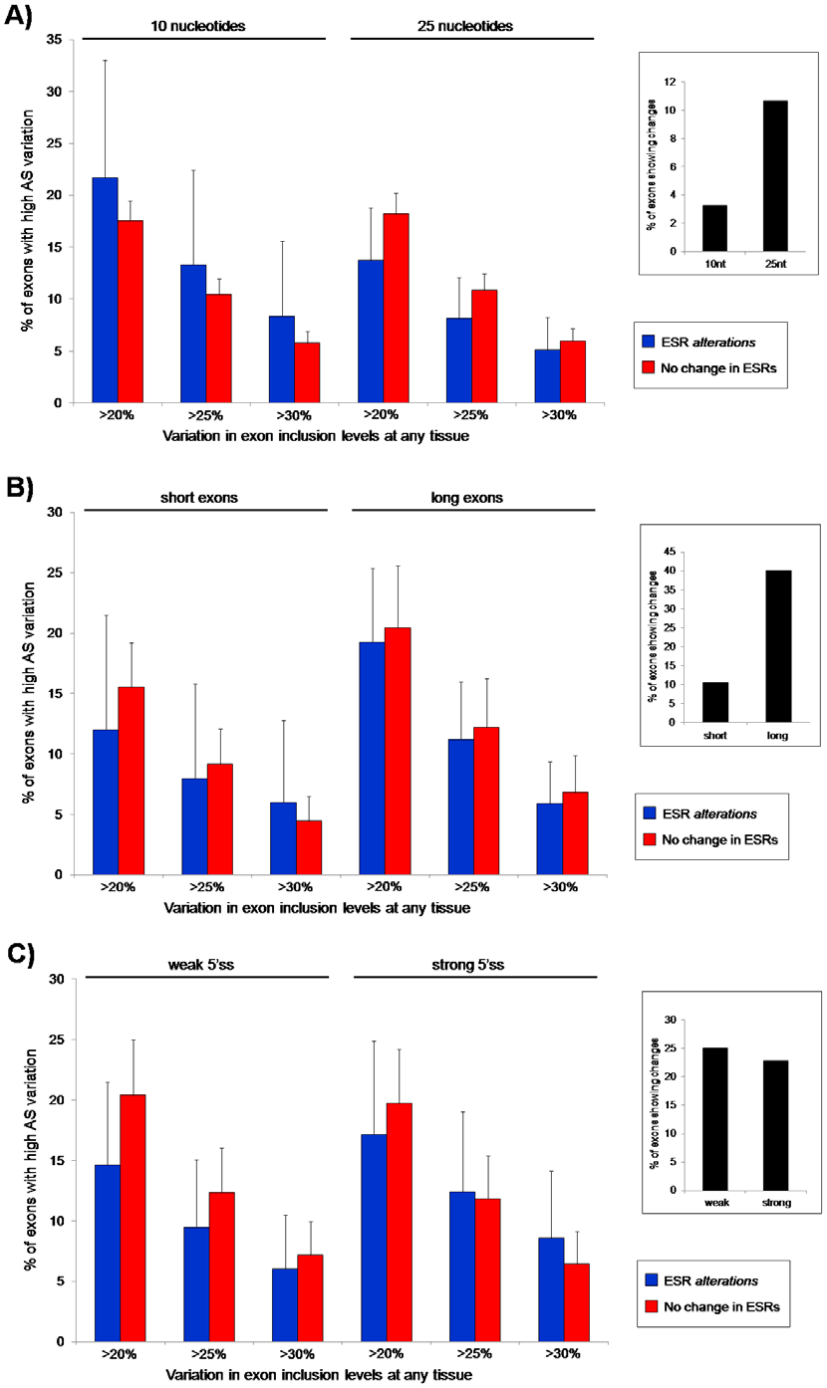
ack of association between ESR changes and changes in AS level at different exon positions and for different groups of exon lengths and 5′ss strengths. (A) Percentage of exons with ESR-altering changes (blue) and without changes in ESRs (red) at the 10 or 25 nucleotides next to the 5′ and 3′ splice sites for different cutoffs of AS variation (y-axis, >20%, >25% or >30% difference in inclusion levels) between human and chimp and datasets. (B and C) Percentage of exons with ESR-altering changes (blue) and without changes in ESRs (red) for short and long exons (B) and weak and strong 5′ss (C) for different cutoffs of AS variation (y-axis, >20%, >25% or >30% difference in inclusion levels) between human and chimpanzee. Right-hand side panels show the percentage of the total exons that have changes in ESRs for the different tests. These results correspond to ESRs from Ke *et al.*'s dataset, and they are similar for the other available dataset and global nucleotide change (data not shown).

Previous studies have shown that regions near boundaries of alternatively spliced exons are enriched in ESRs [29], [41], and that ESRs and synonymous positions in general at these boundaries are usually more conserved than those located in interior regions of exons [29], [42], [43], suggesting greater functional impact of ESRs near exon-intron boundaries. However, we still found no association with AS changes for ESRs located near exon-intron boundaries (within 10 or 25 nts; Figure 3A).

Average exon length and 5′ss strength are known to be different between alternatively and constitutively spliced exons [38], [44]–[47], likely due to alternatively spliced exons having suboptimal spliceosomal recognition signals [48]. Accordingly, conservation of silent sites show differences among exons with different lengths and 5′ss strengths [38]. To address the impact of differences in exon length or 5′ss strength on our results, we divided exons in various groups (see Methods), and studied whether changes in ESR composition affected AS variation in the different groups. For all inclusion level differences and all ESR datasets, we found no differences between short and long exons or between exons with weak and strong 5′ss (Figure 3B, C).

### Changes in ESE versus ESS composition are not predictive of direction of change in inclusion levels

**Figure 4:**
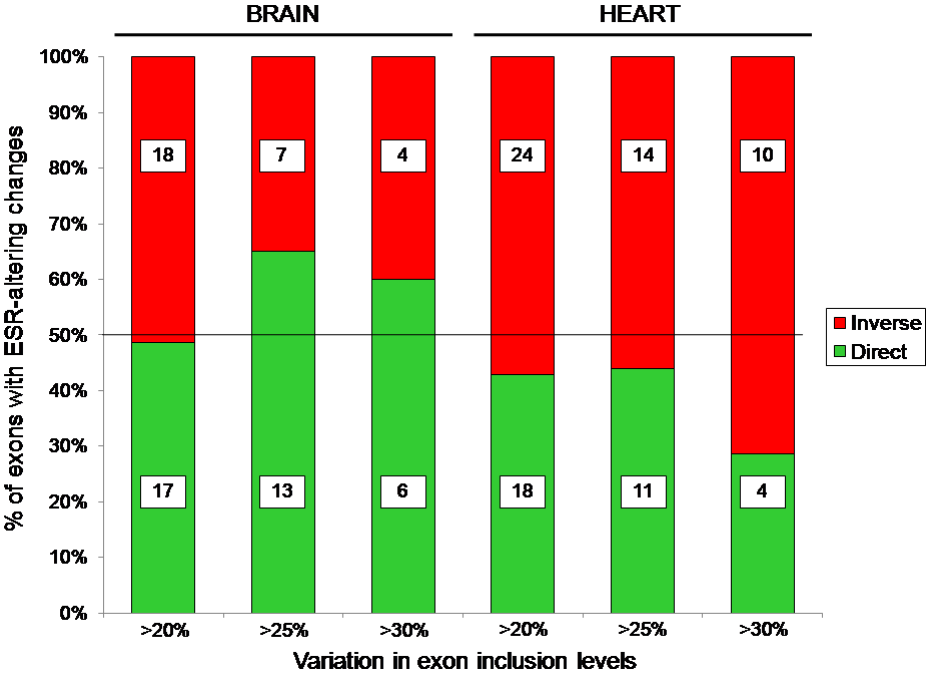
ercentage of exons showing ESR changes and high AS variation at different cutoffs with overall net ESE/ESS composition change consistent with the increase/decrease of exon inclusion level (green) or ‘inverse’ (red). Boxes show the number of exons in each category.

A second prediction of the splicing code model for the evolution of AS inclusion levels is that changes in the composition of ESE versus ESS motifs should be predictive of the direction of differences in inclusion level. That is, an exon with more ESE motifs and/or fewer ESS motifs in one species would be expected to exhibit higher inclusion levels in that species. For each exon with changes in both inclusion level and ESE/ESS composition, we asked whether the direction of the difference in the inclusion level was ‘consistent’ with the expectation from the ESE/ESS difference, or was ‘inverse’. We found that numbers of consistent and inverse changes were similar over a variety of conditions (Figure 4), and often inverse cases outnumbered consistent ones. Thus the character of ESE/ESS changes is not predictive of direction of change of inclusion levels.

## Discussion

The importance of ESRs for splicing regulation is attested to by (i) the preferential occurrence of ESRs in exons, and near exon-intron boundaries; (ii) in vitro modification of AS patterns by introduction or removal of ESRs in reporter minigenes [29]–[31], [34]–[36], [38]; and (iii) the association of naturally occurring nucleotide polymorphism within ESRs with modification of AS, sometimes associated with human disease [49]–[51]. This demonstrated importance of ESRs for splicing predicts an association of evolutionary changes in ESR sequences with changes in splicing patterns. However, we find no association of changes in predicted ESR motifs with changes in AS levels between human and chimpanzee. This lack of association holds across a variety of previously reported ESR catalogs [29], [30], [40], for both studied tissues (brain and heart) individually, for ESEs and ESSs separately, for ESRs located near splice sites, and for exons with different lengths and 5′ss strengths.

What explains this paradox? One possibility is that most observed changes between human and chimpanzee occur in sequences that resemble ESRs, but do not in fact play a role in splicing: although some instances of ESRs have been shown to impact splicing patterns in specific contexts, it does not follow that all instances of an ESR-like sequence have true roles in splicing. Indeed, computational algorithms for identification of ESRs identify motifs that are overrepresented in regions likely to be important for splicing; these motifs are also found in other regions, suggesting that every instance of an ESR-like motif is not a true ESR. The available ESR datasets predict very high densities of ESRs, up to 43.5 ESRs per 100 exonic nucleotides and up to 87.5% of exonic nucleotides potentially involved in at least one ESR (Table1). Considering this high density, it seems unlikely that all ESR instances are in fact bound by *trans*-factors and play an actual role in splicing regulation. Consistent with this argument, previous studies have shown that some changes in ESR-like motifs had no or only subtle effects on the inclusion levels [29], [35], and that these effects are highly dependent on the genetic (gene location, other *cis*-elements) and cellular (presence of specific *trans*-factors) context, having even opposite or no effects when located in different exons or in different positions within the same exon (Figure 4; [26], [28], [29], [41], [52]). Similarly, most predicted binding site instances for specific AS regulators have been shown to be non-functional, despite perfect match to the binding site sequence consensus. For example, most *Fox* binding motifs (UGCAUG) predicted in introns are not functional, particularly those that are not conserved through evolution ([53] and Benoit Chabot, personal communication).

All of these findings point towards the difficulty of identifying true ESRs simply from genomic sequence. In this context, *de facto* ESRs with large effects on splicing are likely to evolve slowly under strong purifying selection [40], [42], [54], [55] whereas ESR-like motifs with smaller or null effects may evolve much more rapidly (close to neutral rates). Therefore, the few observed interspecific changes could primarily occur in ESR-like motifs with little or no effect, thus not leading to changes in alternative splicing levels. Importantly, this effect will be especially noticeable over short evolutionary distances: “neutral” ESR-like motifs will become disrupted relatively rapidly over evolutionary time, towards the limit in which all neutral motifs have been disrupted (analogous to the phenomenon of ‘saturation’ at individual sites); functionally important changes will be slower, and accumulate over longer timescales.

### Implications for studies on human health

Several studies have underscored the potential importance of splicing in human disease, suggesting that SNPs falling within ESRs may be medically important [49]–[51]. The present results underscore the potential downfall of computational screens for SNPs within ESR-like motifs. The current results suggest that the multitude of chimpanzee-human differences found within predicted ESRs are likely to be enriched for ESR-like motifs with little effect on splicing, since true ESRs will be under strong selection. The same factor driving this pattern–namely, selection against those ESR changes that do affect splicing–presumably holds for humans as well. Thus computational scans for SNPs within ESR motifs may instead largely identify SNPs within ESR-motifs with little or no function in splicing.

### The evolution of alternative splicing

What types of changes are driving the divergence between human and chimpanzee in AS levels for a significant fraction (6–8%) of AS exons [15], if not ESR changes? One possibility is that changes in other *cis*-regulatory elements, located in the introns (Intronic Splicing Regulators, ISRs) [56]–[61] or in the upstream and downstream constitutive exons [57] may have more important impacts. However, genome-wide study of ISRs is currently difficult, as less is known about ISRs, and their function seems to be highly context dependent, with position within the intron also strongly restricting the functionality of the elements and determining the effect on splicing of the functional ISRs [52], [53], [62].

A second possibility is that changes in AS patterns across different genes could be driven by changes in a relatively small number of splicing *trans*-factors. Changes in the cellular expression level and/or activity of splicing *trans*-factors [63] can produce widespread changes in exon inclusion levels, and some *trans*-factors do show different expression levels between human and chimpanzee [63]. Changes in the expression of a few *trans*-factors regulating a large number of genes could reconcile the apparent discrepancy between the relatively high level of AS divergence and the low level of sequence divergence between human and chimpanzee (for instance less than a third of the studied exons showed any difference in nucleotide sequence). If this were the case, it would be the presence, and not the change, of some specific ESRs that would be associated with changes in AS level variation. Unfortunately, the lack of sufficient knowledge on most splicing factors' binding sites made it impossible for us to test this hypothesis. For the few proteins for which we have information on binding sites [64]–[66], either there is no comparative data available, or no change has been observed between human and chimp tissue expression [63]. A more general prediction could be that splicing of exons with higher densities of total number of ESRs (and thus more trans-factor binding sites) would be more sensitive to changes in *trans*-factors; however, we found no correlation between exonic ESR density and change in AS levels.

Thus, it seems likely that observed changes in exon inclusion levels between human and chimpanzees are due to a combination of all these causes: changes in few *de facto* splicing regulatory elements and *trans*-factor expression and/or activity.

Finally, another possibility is that the lack of observed association could reflect problems with the data. Experimental noise may affect the results significantly. The rates of false positives and false negatives among both ESR motifs and alternative splicing changes remain unknown [30]. Similarly, despite that RT-PCR validation confirmed 30/37 (81%) tested alternatively spliced exons [15], there may still be a significant fraction of inconsistent quantitative data, as previously reported in other cases of quantification of changes in AS by exon arrays [67]. Finally, the resolution of the quantitative microarray profiling (∼15% difference in inclusion levels) may hide an important fraction of changes in AS levels associated with changes in ESR sequences [29], [30], [35].

### Concluding remarks

In conclusion, we are far from being able to predict the evolution of alternative splicing levels from the evolution of a predicted splicing code. Our results underscore the current difficulty for predicting and understanding human AS regulation solely from sequence evolution. The availability of new high throughput techniques, especially CLIP-seq [41], [52], will improve genome-wide identification of truly functional regulatory motifs, and aid in unraveling the rules governing function of splicing regulators.

## Methods

### Quantitative microarray profiling for human and chimp alternatively spliced exons

Inclusion levels for alternatively spliced exons in two different tissues (brain cortex and heart) for human and chimp were obtained from the supplemental materials of Calarco *et al.*'s [15]; http://www.utoronto.ca/intron/Hs_vs_Pt.html). A total of 1845 alternatively spliced exons were included in this study, 1516 of them expressed in brain and 1534 in heart (1262 expressed in both tissues). The difference in percentage of transcripts including a given exon (‘exon inclusion level’) between the two species was calculated for each exon in each tissue, and used as a measure of change in AS level.

Briefly, these microarray data were generated using custom human oligonucleotide microarrays [15]. Image processing and normalization was done as previously described by Pan *et al.* [68], and confidence-ranked percent inclusion level predictions were obtained from the processed intensity values using GenASAP algorithm [68], [69]. For further information on the methodology, see [15], [68]. Importantly, RT-PCR verification was performed for 37 AS events showing different levels of variation (from no variation to >25% difference in inclusion levels in any or both tissues). 30 of these (81%) showed the expected difference [15]. The resolution of this microarray methodology is ∼15% in exon inclusion differences [15].

### Exonic splicing regulators datasets

In this study we used sets of exonic splicing regulator motifs from three different studies [29], [30], [40], which together comprise nearly all the studies reporting putative ESRs to date.

The first dataset was obtained from Ke *et al.* [40] (K-dataset). In this study, the authors generated a consensus dataset of hexamers from previous studies investigating ESEs and ESSs. The set of predicted ESE hexamers was produced by merging RESCUE-ESEs and PESE (ESE octamers) signals, and the set of predicted ESSs by merging FAS-hex3 ESSs and PESS (ESS octamers) signals ([31], [34]–[36]; these data sets, in turn, were obtained by a combination of computational methods and experimental validation). This yielded 403 predicted ESEs and 199 predicted ESSs in total.

The second dataset was obtained from Stadler *et al*. [30] (S-dataset). In this study the authors designed an algorithm called Neighborhood Inference (NI) that relies on the observation that sites bound by DNA- and RNA-binding proteins tend to cluster closely in ‘sequence space’ (i.e. proteins tend to bind to partially degenerate sequence motifs). They applied this algorithm to a “confident ESE/ESS dataset”, generated from similar sources as in Ke *et al.* [31], [34], [36], containing 666 “trusted” ESE hexamers and 386 trusted ESS hexamers. The use of NI methodology yield an additional 386 ESEs and 100 ESSs using a cut-off score of 0.8 (i.e. a total of 1052 ESEs and 486 ESSs).

The third dataset was obtained from Goren *et al.* [29] (G-dataset). The authors used comparative genomics and dicodon overrepresentation to generate a list of 285 predicted ESRs. Some of these ESRs were experimentally validated using minigen reporter assays under different genetic contexts. Importantly, this dataset does not distinguish between ESEs and ESSs, since the authors show that the effect of ESRs on exon inclusion levels is highly variable and strongly context-dependent [29].

Finally, we built a consensus dataset (C-dataset) with 87 ESRs that were present in all three described datasets.

Consensus binding sites for SF2/ASF (crsmsgw), SRp40 (yywcwsg), and SRp55 (yrcrkm) [64], SC35 (gryymcyr) [65] and hnRNPA1 (tagggw) [66] were obtained from the original sources.

For further information of the methods used to generate the predicted ESR lists, please consult the original sources.

### Analysis of the evolution of ESR signals and effect on AS variation

For each of the 1845 studied alternatively spliced exons we obtained the human and chimpanzee exons as well as the 5′ splice site (5′ss) sequences from UCSC (http://genome.ucsc.edu/cgi-bin/hgTables?org=human), using the Galaxy platform (http://main.g2.bx.psu.edu/), and from Calarco *et al.* (http://www.utoronto.ca/intron/Hs_vs_Pt.html) supplementary materials. The sequences were carefully checked for errors during retrievement.

Each orthologous sequence pair was aligned using ClustalW. For each alignment we studied the conservation for each six nucleotide window (i.e. we studied hexamers beginning at each nucleotide site). For windows with interspecific differences (often only a single substitution), we classified each hexamer as either ESE, ESS, or non-ESR. Based on these classifications, the pair of orthologous hexamers was classified as one of six relationships–ESE/different ESE, ESE/non-ESR, ESE/ESS, ESS/different ESS, ESS/non-ESR, and non-ESR/non-ESR (called ‘neutral’). We then defined total sets of ESE-disrupting changes (ESE/non-ESR+ESE/ESS) and ESE-altering changes (ESE-disrupting plus ESE/different ESE), and analogously defined ESS-disrupting and altering changes. This was carried out for each of the K- and S- datasets. For G- and C-datasets, only changes in general ESR could be assessed: classes of change were thus ESR-disrupting (ESR/non-ESR), ESR-altering (ESR/non-ESR+ESR/different ESR) and neutral (non-ESR/non-ESR).

We studied all overlapping hexamers in each exon. Thus, a single nucleotide change produces changes in 6 consecutive hexamer, which were studied independently. We also used a more strict criterion for ESR change between two species taking into account overlapping hexamers [40]: for any basepair change that introduces a ESE or ESS in one species, none of the corresponding 6 overlapping hexamers in the other species can be an ESE or ESS, respectively.

95% confidence intervals for each group were calculated as in [70] and full Bonferroni correction was used to correct for multiple testing.

### 5′ splice site strength and length subgroup definitions

5′ss strength was calculated using the consensus values score (CV score), as previously described [33], which takes into account positions −3 to +6, i.e. the three exonic positions before and six intronic positions after the splice junction.

We also investigated the possible effect of exon length and 5′ss strength on the relation between ESR evolution and AS variation. For this, we divided the 1845 studied exons into 4 groups quartiles of 5′ss strength or exon length. ‘Weak’ and ‘strong’ 5′ss groups correspond to top and bottom quartiles (CV scores ≤68.49 and ≥78.83); as do ‘short’ and ‘long’ exon groups (lengths ≤5 and ≥146 nucleotides).

## Supporting Information

Figure S1 Lack of association between ESR changes and changes in AS level in brain cortex. Percentage of exons with ESR-altering changes (blue) and without changes in ESRs (red) in brain cortex for different cutoffs of AS variation (y-axis, >20%, >25% or >30% difference in inclusion levels) between human and chimp and datasets. Right-hand side panels show the percentage of the all exons that have changes in ESRs for the different available datasets.
(0.17 MB TIF)

Figure S2 Lack of association between ESR changes and changes in AS level in heart. Percentage of exons with ESR-altering changes (blue) and without changes in ESRs (red) in heart for different cutoffs of AS variation (y-axis, >20%, >25% or >30% difference in inclusion levels) between human and chimp and datasets. Right-hand side panels show the percentage of the all exons that have changes in ESRs for the different available datasets.
(0.15 MB TIF)

Figure S3 Lack of association between ESE and ESS changes and changes in AS level. Percentage of exons with ESE-altering (left) or ESS-altering (right) changes (blue) and without changes in ESRs (red) for different cutoffs of AS variation (y-axis, >20%, >25% or >30% difference in inclusion levels) between human and chimp and datasets. Right-hand side panels show the percentage of the all exons that have changes in ESRs for the different available datasets.
(0.15 MB TIF)
