# Supplementary figures and images for: Evolution of Alternative Splicing Regulation: Changes in Predicted Exonic Splicing Regulators Are Not Associated with Changes in Alternative Splicing Levels in Primates
Source: PLoS One. 2009 Jun 4;4(6):e5800. doi: 10.1371/journal.pone.0005800 (PMC2686173; doi:10.1371/journal.pone.0005800)

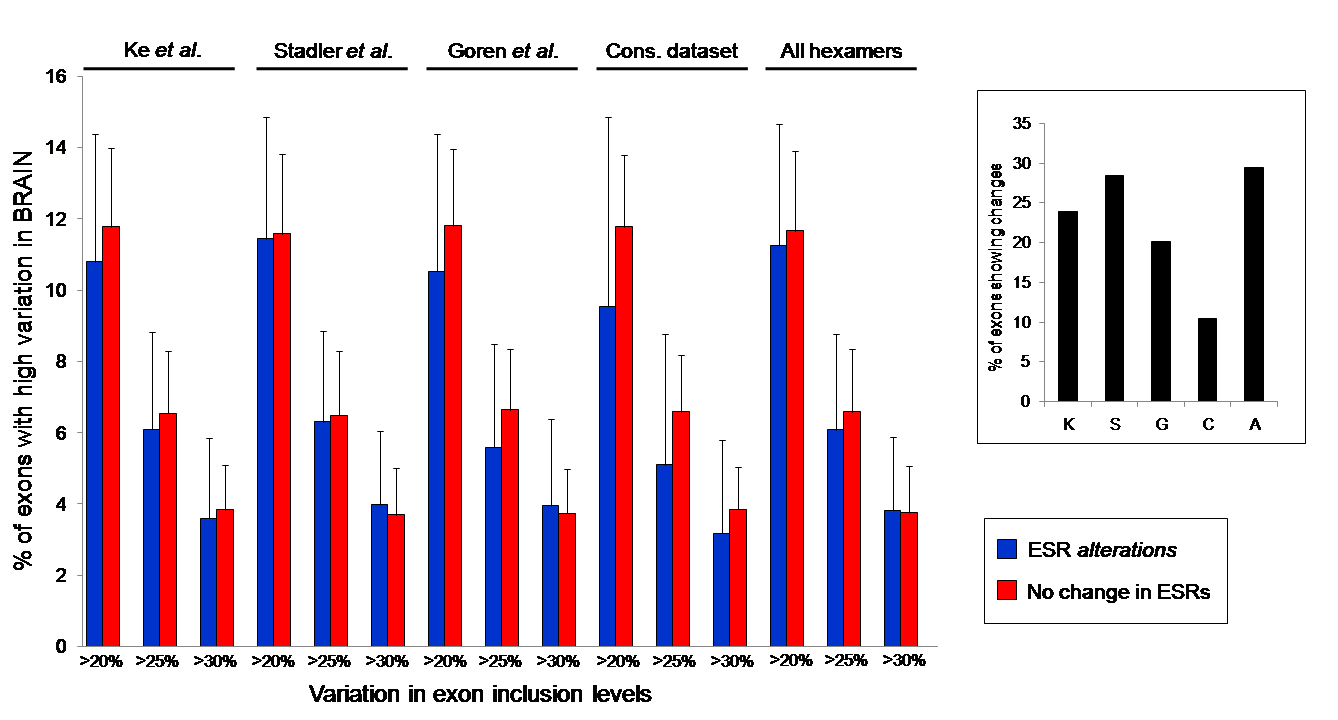

Supplement: Figure S1 — Lack of association between ESR changes and changes in AS level in brain cortex. Percentage of exons with ESR-altering changes (blue) and without changes in ESRs (red) in brain cortex for different cutoffs of AS variation (y-axis, >20%, >25% or >30% difference in inclusion levels) between human and chimp and datasets. Right-hand side panels show the percentage of the all exons that have changes in ESRs for the different available datasets. (0.17 MB TIF) [file pone.0005800.s001.tif]

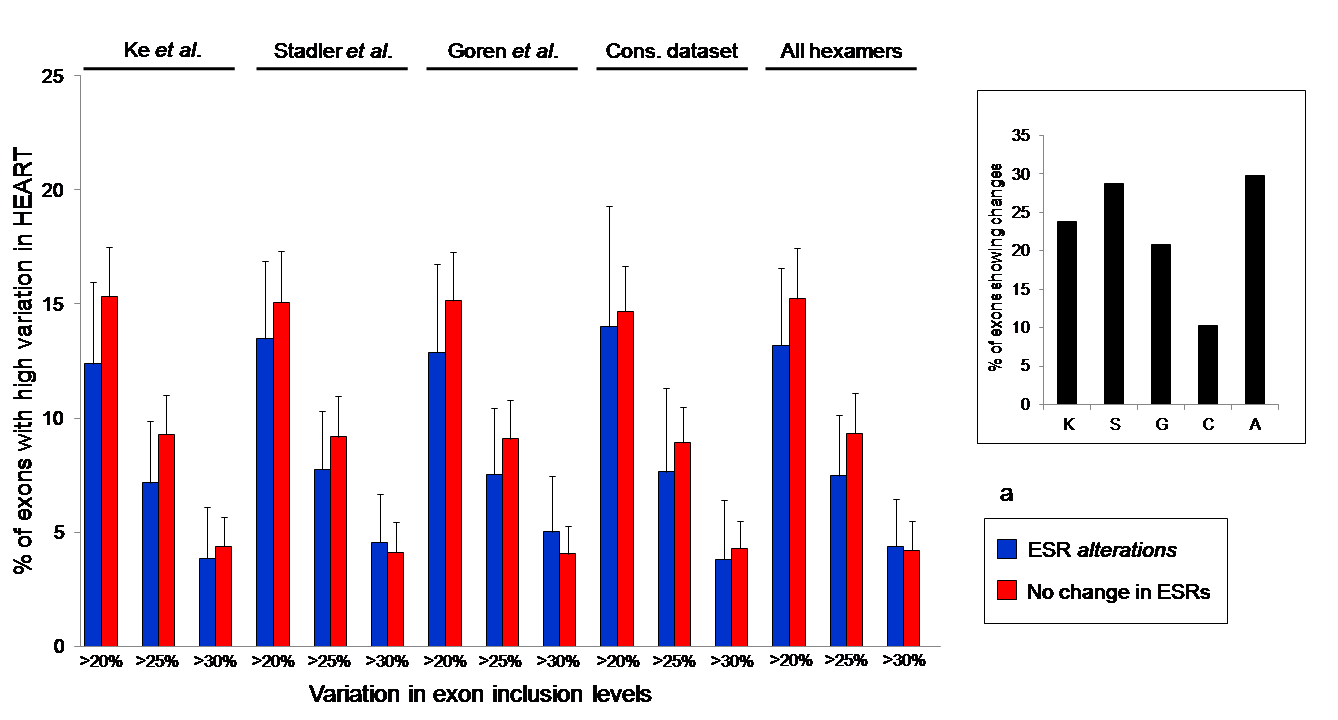

Supplement: Figure S2 — Lack of association between ESR changes and changes in AS level in heart. Percentage of exons with ESR-altering changes (blue) and without changes in ESRs (red) in heart for different cutoffs of AS variation (y-axis, >20%, >25% or >30% difference in inclusion levels) between human and chimp and datasets. Right-hand side panels show the percentage of the all exons that have changes in ESRs for the different available datasets. (0.15 MB TIF) [file pone.0005800.s002.tif]

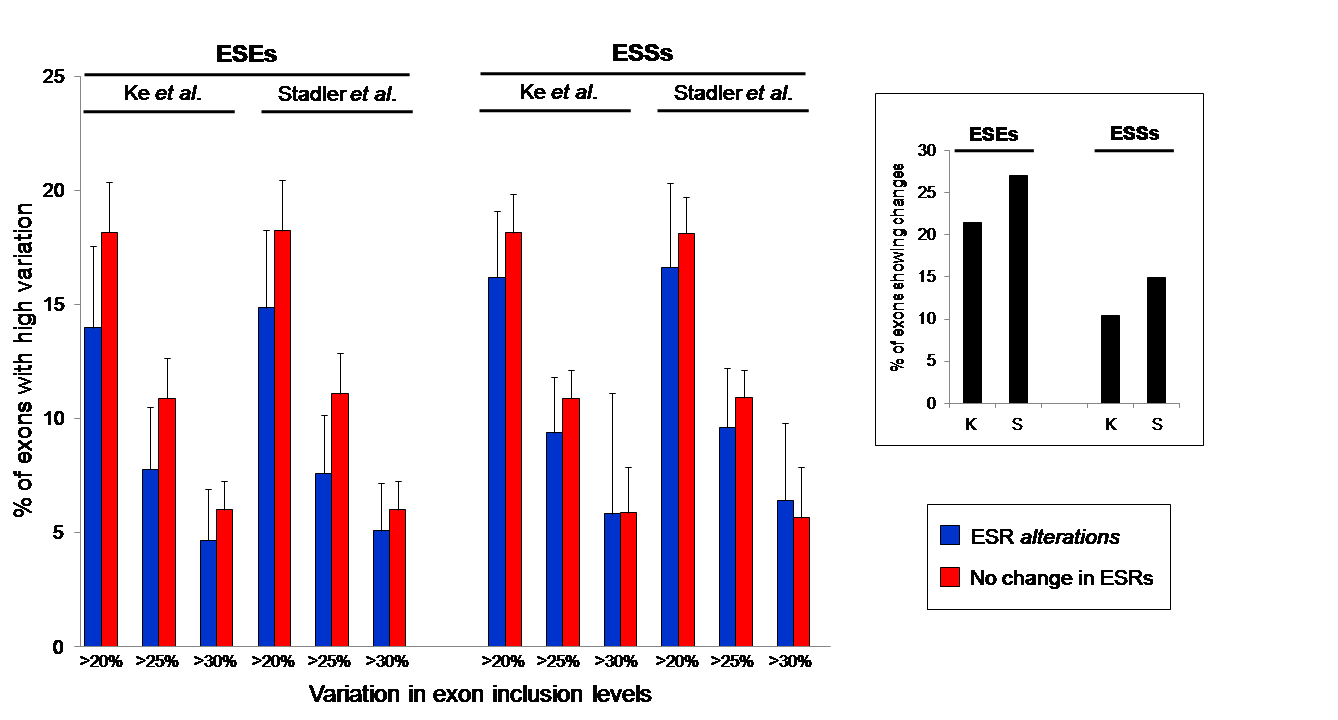

Supplement: Figure S3 — Lack of association between ESE and ESS changes and changes in AS level. Percentage of exons with ESE-altering (left) or ESS-altering (right) changes (blue) and without changes in ESRs (red) for different cutoffs of AS variation (y-axis, >20%, >25% or >30% difference in inclusion levels) between human and chimp and datasets. Right-hand side panels show the percentage of the all exons that have changes in ESRs for the different available datasets. (0.15 MB TIF) [file pone.0005800.s003.tif]
